# Model for managing scientific research in a public hospital: case study: Chilean National Cancer Institute, from 2015–2022

**DOI:** 10.3332/ecancer.2024.1661

**Published:** 2024-01-30

**Authors:** Ximena P González, Isabel Abarca-Baeza, Carmen Gloria San Martin, Ana Belén Ilabaca, Andrea Ibañez-Zuñiga, Rafael Herrada, Berta Cerda-Álvarez, Juvenal A Ríos

**Affiliations:** 1 Scientific Research Center Office, National Cancer Institute, Av. Profesor Zañartu 1010, Independencia, Santiago, Chile; 2 Directorship and Deputy Directorship, National Cancer Institute, Av. Profesor Zañartu 1010, Independencia, Santiago, Chile; 3 Master's Program in Public Health, School of Public Health, University of Chile, Av. Independencia 939, Santiago, Chile; 4 PhD Program in Biomedical Research Methodology and Public Health, Autonomous University of Barcelona, Plaça Cívica, 08193 Bellaterra, Barcelona, Spain; 5 Directorship of Translational Medicine Program, University Mayor, 8580745 Huechuraba, Región Metropolitana, Chile

**Keywords:** research, hospitals, cancer, health, translational medicine, oncology

## Abstract

Research is an essential element in the practice of healthcare, and hospitals play a fundamental role in its promotion. Research in hospitals can improve the quality of care, knowledge of diseases and the discovery of new therapies. Hospitals can conduct research in various fields, including basic research, clinical research, population-based research and even hospital management research. The findings of hospital research can be directly applied to clinical practice and management, thereby enhancing the quality of patient care, a central paradigm in translational health. This article details the experience of the National Cancer Institute of Chile over the past 8 years in its role as a high-complexity public hospital, specialised institute, healthcare centre, teaching institution, and research facility. It reviews the work of generating and strengthening its institutional research model since its redesign in 2018, the key elements that underpin it, and discusses the challenges the institute faces in its growth amidst the increasing cancer epidemiology in Chile, the recent enactment of a National Cancer Law, the post-pandemic scenario that has left a significant waiting list of oncology patients, and the initiation of the design and construction process for the new institute building.

## Basis and historical background

Health research is fundamental for the continuing improvements in medical care, prevention, and treatment of disease. In said efforts, hospitals are essential in that they are the locations for clinical care, as well as generating knowledge and promoting research projects. The National Institutes of Health, National Health Service, and the Centres for Disease Control and Prevention are examples of institutions that work, among other things, to promote health research, through financing and other strategies that contribute to hospital participation in research [1–4].

There are various reports that demonstrate thoroughly how hospital work contributes positively to health research [5]. Hospitals are considered to be places of confluence and interaction with universities, covering various areas of knowledge, such as basic sciences, for example. For their part, hospitals provide services from diagnosis and supplying medication, to clinical care and direct links to patients. Needless to say, hospitals are where the majority of clinical trials are performed; therefore, the creation of new medications occurs there [5].

Health professionals are faced with daily challenges, which give rise to relevant clinical and epidemiological research questions. Therefore, hospitals may themselves be incubators for scientific and technological ideas [6, 7]. However, so that those ideas may be channelled and transformed into concrete applications for patient benefit, it is necessary to create a structure, resources and an *ad hoc* team, as suggested by the available scientific evidence [8].

Globally, hospital offices for Research and Development (R&D) are emerging as a paradigm for these care centres [8]. These offices are composed of a multidisciplinary team of professionals capable of providing administrative and methodological support for the ideas suggested in the hospital. Thus, they seek to promote a ‘culture’ of research within the care centre [8, 9]. In Chile, some experiences have been implemented, with differing levels of development and success. One of these is the Institute of Maternal and Child Research (Instituto de Investigaciones Materno Infantil), established in 1988 and housed in the ‘San Borja Arriarán’ Public Hospital. This institute has sustained itself over time, and combines clinical practice with research in areas such as reproductive medicine, infertility, and genetics, among others [10]. Other experiences have not sustained themselves over time, such as the case of the R&D program in the ‘El Carmen’ Hospital in Maipu, which, despite this, succeeded in carrying out research in epidemiology, physical medicine/rehabilitation, and applied neuroscience between 2015 and 2018 [11].

The case of the Chilean National Cancer Institute (Instituto Nacional del Cáncer de Chile (INCANCER)), dates to 2005, although there were previous initiatives recorded in the book ‘History of the National Cancer Institute: a challenge in the fight against a millennial disease’ [12]. In 2005, a supreme decree was established, urging self-managed establishments to have a ‘policy for research in their specialty’. One year later, in 2006, ICANCER became the premier self-managed network establishment, according to Exempt Resolution No. 368 of 11 March of 2006 [13].

In 2007, the Centre for Responsibility (CR) in Research and Teaching was established in INCANCER, upon which the Scientific Research Unit depends. Review committees for Clinical Studies and the evaluation of the use of Research Funds were also created. In 2012, the decision was made to separate the functions of Research and Teaching; creating the CR in Research and Teaching as independent entities. Both CRs are advisory entities and dependent upon the Medical Directorate of the Institution; however, in 2015, they became dependant upon the Medical Deputy Directorate of Institutional Development (Sistema de Monitoreo del Desempeño Institucional (SMDI), in Spanish). Since 2007, the Scientific Research Unit has been charged with the responsibility for registration, coordination, monitoring, and financial control of the scientific research activities of the establishment.

Between 2017 and 2019, together with the Hospital Pre-investment Study (EPH, in Spanish) to provide INCANCER with new infrastructure and resources (BIP30372776-0) [14], work was revitalised around the central principle of research as one of the three pillars of the mission and vision of the institution, accepting itself in the model of projected management, and defining its resources so that the new Institute can fulfil its role in this aspect. The hospital investment project represents a crucial moment for the development of the Institute’s current research model. During 2018, human resources were introduced and a redesign process was begun, laying the foundation for the future Institute as a centre of excellence in cancer research.

During this time, significant advances were realised in the normalisation and organisation of core aspects of research activity; including the reactivation and restructuring of the institutional research committee (RC), the redefinition of conditions for the development of research projects in collaboration with the industry, and the implementation of the first steps of the institutional financial management model. In addition, processes were identified and defined to provide assistance and support to the research initiatives involving the institutional community and patients’ participation, along with strategic alliances with diverse academic entities.

At the end of 2018, the country was immersed in the development of a National Plan for Cancer that also promoted a National Law on this subject [15–17]. Both initiatives included the participation and leadership of professionals from the Institute. In fact, the doctor working as acting Director at INCANCER at that time stepped down from this position to take on a leading role at the Cancer Department of the Ministry of Health. After reorganising the Centre for Research Accountability (Centro de Responsabilidad de Investigación (CRI)) in 2018, teams were improved by incorporating professionals skilled in areas such as project management, clinical studies and good clinical practices, among others. This strengthening of the team allowed improvement of the Institute’s capacities and the addressing of the challenges related to research activities in a more integrative, systematic and regulated manner. It is worth mentioning that in 2021 the health authority confirmed the Institute’s role as a complex care centre that ‘should also play a role in teaching and education, as well as research and knowledge production for cancer management in the country’, as stated in ORD.C26 N.° 998 from 13th April 2021.

Moreover, the CRI’s purpose is manifested in the latest version of the organisation and functions manual from 2022. This internal-use manual states that the CRI’s purpose is to promote scientific research and innovation within the institution in order to improve cancer patients’ health. It should be pointed out that this document is based on various national regulations, which provide solid regulatory support [18–21].

This paper presents the National Cancer Institute’s experience in boosting their role as a public centre conducting cancer research. It addresses key aspects related to the institute’s governance, interactions across institutions and with the community, funding sources and its main achievements so far. Lastly, its main challenges are also described.

## Management model

### Governance

The responsibility of managing the institutional research model lies with the CRI, which answers to the Monitoring System of Institutional Performance SMDI. This team coordinates the RC (Comité de Investigación (CI)), which consists of representatives from various clinical and administrative areas. It is worth mentioning that the CI is an advising body to the leadership in this area, and provides recommendations including long-term strategic guidelines, regardless of the current administration at CRI. One of the CI’s roles is to suggest approval or refusal of the execution of research projects at the Institution.

**Figure 1.**
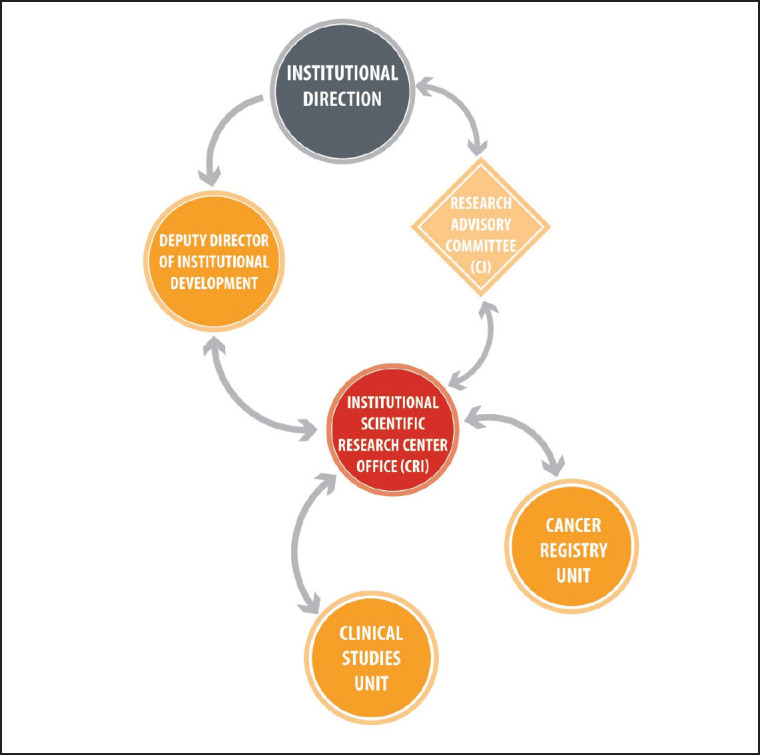
The CRI organisational chart. The CRI depends on the Institutional Development Sub-Division, which, in turn, is a direct dependency of Institutional Management. Simultaneously, the CRI presents the projects prepared by the researchers before the RC, which is not part of the hierarchy of the institution itself, but is an advisory body. It is important to highlight that, up to the date of this publication, the CRI is made up of two units: that of clinical studies and the cancer registry. The professionals involved in the development of the model since 2018 and as of the date of this publication are: Institutional Management (2018 Sergio Becerra P.; 2019 to present Berta Cerda A., Institutional Development Deputy General Manager, Isabel Abarca B.; CR Research 2018, Isabel Abarca B., 2019 to present, Ximena González S.; Clinical Studies Unit, Carmen Gloria San Martin C.).

At present, the CRI’s leadership has a technical and administrative background, and reports its actions and results to the SMDI, which answers to the Institute’s directors. Under the CRI’s leadership, there are two units: clinical studies and cancer register (Figure 1). The first unit’s mission is to promote and support clinical research studies and trials related to anticancer drugs, devices and diagnostic methods. The second unit’s role is to gather, systematize and create accurate epidemiological information about the associated cancer population in order to take institutional decisions based on that knowledge. Furthermore, the registration unit should support the development of research initiatives or the identification of questions based on the data. It is important to mention that the cancer register is a cross-sectional team which was created with the establishment of the hospital cancer registers as a ministry’s strategy, in which the team was an integral part during the last decade.

### Internal interactions

**Figure 2.**
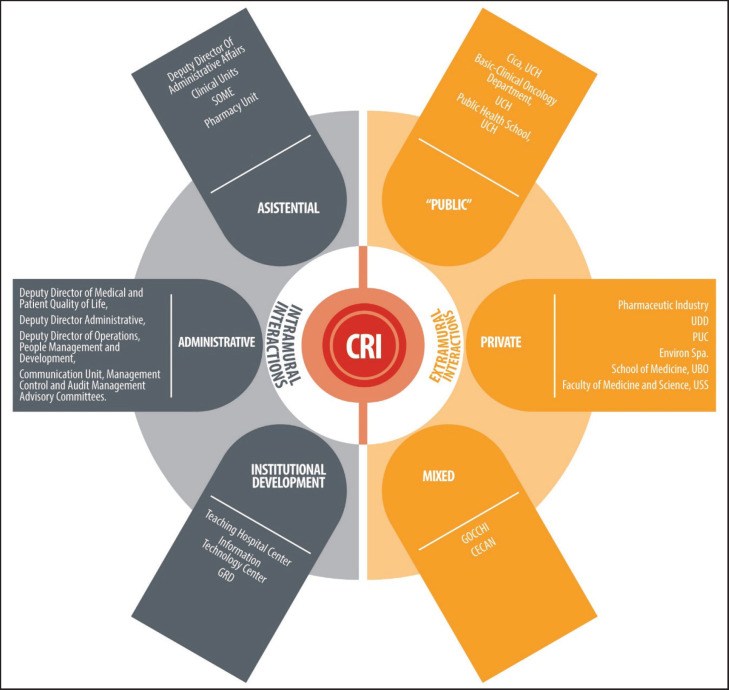
The CRI and its ecosystem of interactions. CICA: Advanced Clinical Research Centre, UCh: University of Chile, UDD: Development University, PUC: Clinical Hospital, Catholic Pontificia University of Chile, UBO: Bernardo O´Higgins University, USS: San Sebastián University, GOCCHI: Chilean Co-operative Oncology Research Group, CECAN: Cancer Prevention and Control Centre, GRD: Related Diagnosis Groups, SOME: Medical Statistical Guidance Service.

Within the institution, the CRI works alongside all sectors at INCANCER, that is, all care, administrative and institutional development units. The main goal of this interaction is to establish a clear flow to lead scientific ideas emerging from the teams and the associated population’s needs, as well as ensuring the CRI’s methodological support to present them to the above mentioned entities, such as the CI and the institute’s directors (Figure 2).

### External interactions

The CRI is associated to public, private and mixed external entities. With each of them, the main goal is to create health knowledge that can be used to benefit cancer patients. The main products from this relationship consist of research projects, published materials, seminars for the dissemination of scientific knowledge and human capital training. Figure 2 shows the organisation of such areas

### Sources of funding

The CRI’s funding primarily comes from two sources: the institution and the private sector. The institution’s funding is used for infrastructure and the unit’s operative expenses (including the CRI’s team wages, infrastructure, supplies, etc.), and is the cornerstone for this division’s operation. The private funding primarily comes from studies sponsored by the pharmaceutical industry and initiatives from companies or science-tech entities developed in an academic institution or spin-off, which enables a model for reinvesting in research for the Institute itself. Furthermore, the state agencies’ funding comes from financial instruments that are government-backed and managed by the National Agency for Research and Development (Agencia Nacional de Investigación y Desarrollo). It is worth noting that, due to administrative reasons beyond the management of INCANCER, so far the institute has not been able to access these funds as a beneficiary institution, but it was able to access them as an associated entity in multiple initiatives. However, efforts are being made and advocacy done to reverse this situation and the Institute, as a research centre, may be subject to financing through these means, as established in Articles 33 and 37 of the organic regulations of less complex health establishments and network self-management establishments [13].

### Flow of a study at the institute

**Figure 3.**
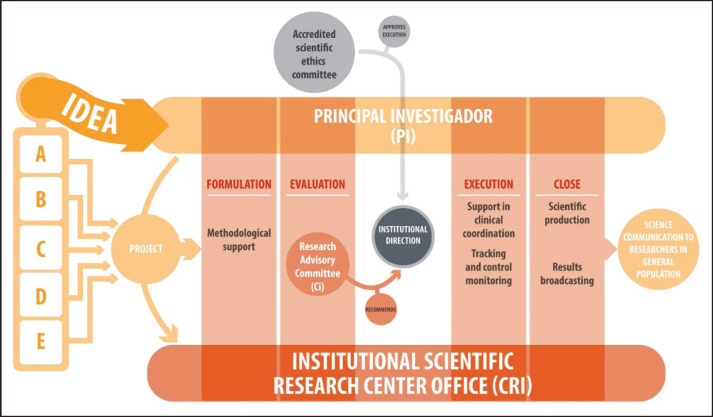
Flow of research ideas at the National Cancer Institute of Chile. A: PI corresponds to a health professional from INCANCER who has a new idea to carry out at the Institute, B: PI corresponds to a health professional from INCANCER who is affiliated with a University and wishes to expand his/her study at the Institute, C: PI corresponds to a University academic, without INCANCER affiliation, who wishes to develop his/her research at the Institute, D: PI is sought by the CR at a University to develop a project of interest to the Institute. E: PI corresponds to a health professional from INCANCER, who is sought by an external institution (e.g., pharmaceutical) to develop his/her study at the Institute. RC: INCANCER internal committee which evaluates the technical relevance of the project and if the research is of corporate interest to the Institution, it does not evaluate the ethical aspects. Accredited Scientific Ethics Committee (ASEC): External committee, (generally from the health service, not from INCANCER), evaluates only the ethical aspects of the research.

The requirements and procedures for the evaluation involving human beings and other research projects are detailed in the internal document: ‘Procedure for the Evaluation of Research Involving Human Beings and other studies, Exempt Res N°002442’. Generally, any study submitted to the CRI must meet a series of specific requirements, regardless of whether it is industry-sponsored studies, internal projects or with external alliances. Once the CRI receives the study, it is forwarded to the CI for evaluation. As observed in Figure 3, the CI will evaluate the project, and recommend (with major or minor observations) or not, its execution, considering institutional priorities and guidelines, resources and impact on care processes and mainly on patients. Similarly, the PI must present the project to an accredited Scientific Ethics Committee and provide the corresponding approval to the CRI so that the institutional management may authorise the project and the researcher may begin their study.

## Products

The scientific research process has been described by Michael Faraday in three simple steps: first, begin it; second, finish it; and third, publish it [22]. To reflect the productivity of the institutional research model, the following products have been defined; publications with INCANCER affiliation, projects and their management, and training.

This work examines the period between 2015 and 2022, during which, according to information recorded in the CRI, a total of 37 articles were published, which represents an average of 5.2 articles per year. It is important to consider two different sub-periods: 2015–2017 and 2018–2022. As seen in Table 1, the second sub-period coincides with the implementation of the current institutional research model; in which a 211% increase in the productivity of publications was observed in comparison to the first. It is necessary to specify that the registered publications may correspond to projects from previous periods, to those contained in Table 2.

With regard to the projects, a total of 251 were registered during the entire period, which is equivalent to an average of 31.3 projects per year (51 in 2015–2017; 201 in 2018–2022), which reflects a percentage increase of 294% in the number of projects with respect to the first period (Table 2). The status of these projects as of 2023 is described in Table 3.

In terms of training, it is important to mention that in collaboration with the CR Teaching and the University of Development, there has been an historic participation in the General Oncology Diploma course through the subject of Research Methodology, which is co-ordinated from the CRI. Likewise, best practices clinical courses have been promoted (already in a third version), and more recently a first version of a scientific writing course in alliance with the Bernardo O’Higgins University School of Medicine aimed at research leaders and the Institution teams.

Additionally, we must take into account that, as part of the products, there is a slight increase in internal revenue generated by CRI activities. This has resulted in the hiring of new positions for its operation, as well as obtaining a percentage of the financing of the clinical trials themselves, which, as already mentioned, have been reinvested in research.

## Conclusions and future challenges

In this work, we have revised the first period of operation of the CRI, its management model, the internal organisation process which has allowed the establishment of an operation base and its relationship with different hospital levels and the environment. In addition, we have confirmed that there is quantifiable productivity in terms of publications, projects and training, which demonstrates a work that is just beginning and that needs solid support, not only from local authorities, but also from central and governmental agencies.

One of the main future challenges is to prioritise the lines of research which the institution will promote hereafter and do it in a participatory way, space which must be given as part of the institutional strategic planning process which began this year, 2023. This involves making decisions about the vision of our institution, internally aligning the priorities and lines of research to focus on our development, at the same time that we adjust to the guidelines of public policy on cancer [23]. It is essential that these lines of research address the most urgent needs of the population in terms of health and cancer and that they are in line with the National Cancer Law, the National Science Policy, Technology and Innovation 2020 and the strategy proposed by the National Innovation Council [17, 24, 25]. Beginning a process of measuring the impact of research for the benefit of patients is pending.

A second obvious challenge, taking into account the high demand for assistance from clinical teams and diagnostic units, is that it is essential that the CRI has in the near future, specialised support in the development of projects and writing of scientific manuscripts.

A third challenge is to ensure long-term financial sustainability. Although it is not an immediate priority, the current CRI financial model must be strengthened. In order to achieve this, it is necessary to explore new sources of income, such as fundraising, to establish alliances with foundations, to consider philanthropy and study and manage certain restrictions or regulatory elements which have been generally installed at the level of the administration of public services and that are not considered exceptional situations such as health research and its financing methods. All this within the framework of health sector reform and the need to ‘think outside of the box’. These are only some of the ideas which ought to be considered to promote the financial sustainability of the research model in a self-managed network establishment.

Lastly, and taking into account the stage which the execution of the investment project of the new National Cancer Institute is at, with launch projected for 2030, it is imperative to jointly build a virtuous ecosystem of sustainable and scalable collaboration in the time. To do this, the CRI must connect with representatives from various sectors, including academies/universities, civil society/patient foundations, the private sector/pharmaceutical industry, state institutions such as the Ministries of Health and Science, and even political actors (Figure 3). Ultimately, our hospital is notable not only for its dedication to healthcare, but also for its focus on teaching and research. These aspects are fundamental for an institute such as INCANCER, which is projected as an essential pillar in the national cancer network. This incorporation of efforts and resources will allow us to advance in the fulfilment of our mission effectively and for the benefit of patients and society as a whole.

## Conflicts of interest

None of the authors present a conflict of interest for this publication.

## Funding

This work did not receive financing.

## Figures and Tables

**Table 1.**
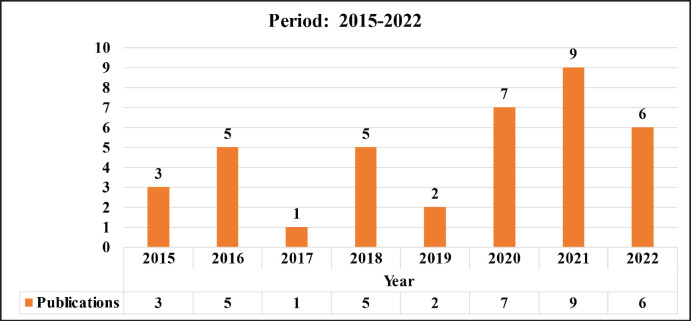
Publications in the period 2015–2022.

**Table 2.**
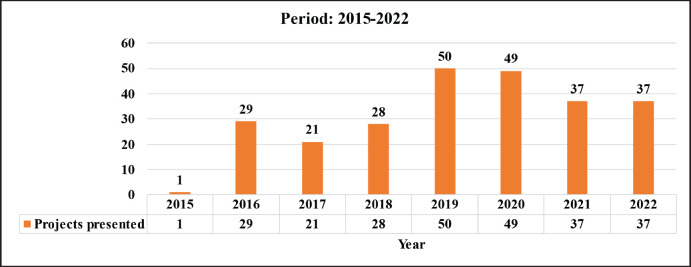
Projects presented in the period 2015–2022.

**Table 3.**
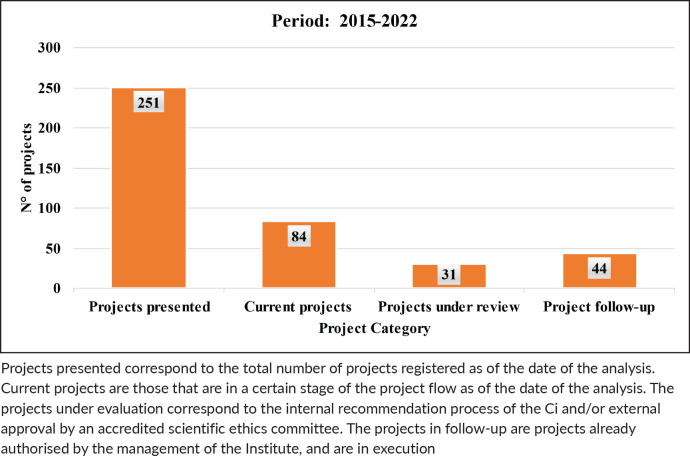
Table 3. Status of projects in the period 2015–2022.
